# RCCS Bioreactor-Based Modeled Microgravity Affects Gastric Cancer Cells and Improves the Chemotherapeutic Effect

**DOI:** 10.3390/membranes12050448

**Published:** 2022-04-21

**Authors:** Nina Rembiałkowska, Dagmara Baczyńska, Magda Dubińska-Magiera, Anna Choromańska, Katarzyna Bieżuńska-Kusiak, Agnieszka Gajewska-Naryniecka, Vitalij Novickij, Jolanta Saczko, Dawid Przystupski, Julita Kulbacka

**Affiliations:** 1 Department of Molecular and Cellular Biology, Faculty of Pharmacy, Wroclaw Medical University, Borowska 211A, 50-556 Wroclaw, Poland; nina.rembialkowska@umw.edu.pl (N.R.); dagmara.baczynska@umw.edu.pl (D.B.); anna.choromanska@umw.edu.pl (A.C.); katarzyna.biezunska-kusiak@umw.edu.pl (K.B.-K.); agnieszka.gajewska-naryniecka@umw.edu.pl (A.G.-N.); jolanta.saczko@umw.edu.pl (J.S.); 2 Department of Animal Developmental Biology, Faculty of Biological Science, University of Wroclaw, Sienkiewicza 21, 50-335 Wroclaw, Poland; magda.dubinska-magiera@uwr.edu.pl; 3 Institute of High Magnetic Fields, Vilnius Gediminas Technical University, 03227 Vilnius, Lithuania; vitalij.novickij@vgtu.lt; 4 Department of Pediatric Bone Marrow Transplantation, Oncology and Hematology, Wroclaw Medical University, Borowska 213, 50-556 Wroclaw, Poland; dawid.przystupski@student.umed.wroc.pl

**Keywords:** microgravity, drug resistance, gastric cancer, doxorubicin, cytoskeleton

## Abstract

(1) Background: The main purpose of the study was to determine whether altered gravity might alter cell viability, improve drug delivery and modulate the expression of drug resistance-related genes. (2) Methods: This study investigated the intracellular mechanisms activated by microgravity in human resistant and sensitive gastric cancer cells (EPG85-257 RDB) and (EPG85-257 P). We used a rotary cell culture system (RCCS) developed by NASA to expose cells to altered gravity. The antitumor potential of microgravity was simulated by the RCCS bioreactor, and its effectiveness was evaluated in sensitive cell lines compared to chemotherapy-resistant cells concerning drug-sensitive cancer cells. Microgravity with chemotherapy was estimated by the viability assay, cytoskeleton imaging, MDR (multidrug resistance) gene expression analysis, MTCO-1 (mitochondrially encoded cytochrome C oxidase I), and 8-OHdG immunocytochemical analysis. (3) Results: We found that altered gravity combined with doxorubicin was cytotoxic to cancer cells. Cells following simulated microgravity revealed decreased expression of genes related to drug resistance and increased DNA/RNA damage marker expression. Cytoskeleton evaluation demonstrated significant reorganization of F-actin fibers after exposure to changed gravity conditions. (4) Conclusions: Intracellular alterations caused by simulated microgravity can increase gastric cancer cells’ sensitivity to chemotherapy. We have obtained satisfactory results showing the correlation between altered gravity and MDR phenomena which seems promising in future therapeutic applications.

## 1. Introduction

Changes in gravity produce biological effects, causing alterations in cell morphology, proliferation, and gene expression [1,2,3,4,5]. Numerous studies on simulated Earth microgravity in laboratory conditions observed its biological effect on cells, tissues, and organs. In particular, cancer research has employed microgravity for studying mechanisms that control cancer cell growth and function [6,7]. Thus, there occurred an idea that changes in gravity can be used to modulate and control biological processes. However, only a few works show the effects of microgravity on the response of cancer cells to chemotherapeutic drugs [8,9]. The modulation of cell membranes and the induction of drug resistance mechanisms via microgravity seem a promising alternative for cancer cell elimination [4,10]. The multidrug resistance (MDR) phenomenon is one of the most limiting factors in anticancer protocols. Certain types of cancers, including gastric, colon, pancreatic, and ovarian cancer, overexpress MDR proteins on the cell membrane surface, which causes limited drug delivery [11,12]. Cancers related to the gastrointestinal (GI) tract are one of the most resistant to chemotherapy and often demonstrate primary resistance [13,14]. It was also revealed that GI cancers develop chemo-resistance within the chemotherapeutic sessions, which causes ineffective therapy [15]. The available literature points to different cellular mechanisms that can be responsible for the MDR phenomenon, e.g., mutations in the p53 gene, inadequate drug metabolism, pharmacokinetic resistance [15], overexpression of MDR proteins and genes, such as ABC transporters [16], inactivation of apoptosis signaling pathways, changes in cell cycle checkpoints, and deregulation of noncoding RNAs (ncRNAs) [14]. Thus, developing new and effective protocols to overcome or modulate MDR mechanisms is strongly needed. Hence, physical methods to sensitize or reorganize cell membranes can be an efficient tool for drug delivery. In this study, we intend an application of altered gravity to affect cells’ MDR mechanisms and increase drug delivery and activity. Here, we focused on evaluating cellular mechanisms induced by the altered gravity, i.e., microgravity with simultaneous treatment with doxorubicin in human gastric cancer cells sensitive and drug-resistant in vitro. The cellular alteration associated with the MDR phenomena is revealed in morphological, cytoskeletal, and genetic changes related to the exposure to microgravity. Thus, we believe that this study will provide valuable knowledge for further research in explaining the role of microgravity in cancer therapy.

## 2. Materials and Methods

### 2.1. Cell Cultures

The studies focused on elucidating human gastric cancer cells: daunorubicin—sensitive EPG85-257P and daunorubicin resistant EPG85-257 RDB, obtained as a kind gift from Prof. Herman Lage. EPG85-257 RDB cell line indicates resistance to daunorubicin and overexpression of MDR1 (known as P-gp) mechanism involvement [1]. Gastric cell lines were grown in Leibovitz L-15 medium (Sigma-Aldrich, Poznan, Poland) supplemented by 10% fetal bovine serum (FBS, Lonza, Celllab, Warsaw, Poland), 1% of antibiotics (penicillin/streptomycin, Sigma-Aldrich, Poland), 1 mM ultraglutamine (Sigma-Aldrich, Poznan, Poland), 6.25 mg/L fetuin (Sigma-Aldrich, Poland), 2.5 mg/mL transferrin (Sigma-Aldrich, Poznan, Poland), 0.5 g/L glucose, 1.1 g/L NaHCO_3_, 1% minimal essential vitamins (Mem-Vit, Sigma-Aldrich, Poznan, Poland). The cells were maintained in a humidified atmosphere at 37 °C and 5% CO_2_ (SteriCult, Thermo Scientific, Alab, Warsaw, Poland). For the experiments, the cells were removed by Trypsin-EDTA 0.25% solution (Sigma-Aldrich) and washed with PBS (phosphate-buffered saline) (BioShop, Lab Empire Poland, Rzeszow, Poland).

### 2.2. Simulated Microgravity Protocol

**Figure 1:**
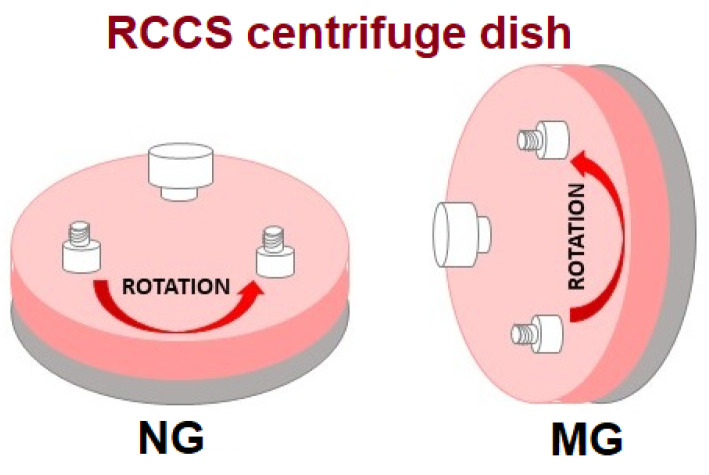
RCCS centrifuge: NG—normal gravity (1 g) and subsequent, MG—simulated microgravity conditions.

Figure 1 shows a diagram of the RCCS (Rotary Cell Culture System) centrifuge used in the research and its positioning. The centrifuge is equipped with a rotating vessel with a volume of 10 mL, each filled with a suspension of cells (5 × 10^5^) in Leibovitz-15 medium with all additives described here [17]. The following rotation conditions were used for normal (NG) and microgravity (MG): 20 rpm and exposure times 24, 48, 72, and 96 h. Independent control cells were prepared and spun under normal gravity (1g-NG) at 37 °C. The behavior of human gastric cancer cells under the influence of disturbed gravity in combination with doxorubicin (0.5 ng/mL = 9.1993 µM) was assessed. For the immunocytochemical, immunofluorescent studies, and gene expression, cells underwent 72 h of centrifugation, and then the changes were assessed.

### 2.3. Presto Blue Viability Assay

Cell viability was examined with a PrestoBlue™ Cell Viability Reagent (ThermoFisher, cat. No. A13262). The viability testing was performed after the exposition to normal (NG) and simulated microgravity (MG), in a rotating vessel (RCCS) for 72 h with or without doxorubicin. Then cell suspension was taken and disposed into the bottom of two wells of the black, 96-well plate with a transparent bottom. Cells were suspended in the growth medium, the PrestoBlue reagent was diluted 1:9 with a culture medium, and 100 µL of the solution was added to each well. After 30 min of incubation at 37 °C, the solution was gently mixed. The fluorescence was detected using a GloMax^®^ Discover Microplate Reader (Promega, Waltham, MA, USA) with a green excitation filter (520 nm) and 580–640 nm emission filter.

### 2.4. Visualization of F-Actin by Confocal Microscopy Imaging

Cells underwent protocols as described in the Section 2.2. Then cells were seeded on cover microscopic slides. After 48 h, cells were fixed for 10 min with 4% PFA (paraformaldehyde) and washed with PBS, and staining was performed according to the manufacturer’s protocols. Actin filaments were stained with Invitrogen™ Alexa Fluor™ 546 Phalloidin (2 µg/mL, Thermo Fisher Scientific, A22283, Waltham, MA, USA) with the producer’s standard protocol. DAPI was used for nuclei staining. FluoView FV1000 confocal laser scanning microscope (Olympus, Tokyo, Japan) was used to visualize actin filaments in human resistant and sensitive gastric cancer cells after normal and simulated microgravity.

### 2.5. Assessment of MDR Genes Expression by Real-Time PCR

After 72 h of centrifugation, the cells were rinsed with PBS and centrifuged (280× *g*, 5 min). The dry cell pellet was stored at −20 °C for further experiments. The total RNA was isolated using a NucleoSpin RNA II kit (Macherey-Nagel GmbH&cCo, Düren, Germany) following the manufacturer’s protocol. Reverse transcription reaction (RT) was performed using 600 ng of extracted total RNA and a High-Capacity cDNA Reverse Transcription Kit (Thermo Fisher Scientific, Waltham, MA, USA) in a final volume of 20 μL according to the manufacturer’s instructions. AceQ qPCR Probe Master Mix (Vazyme Biotech, Nanjing, Jiangsu, China) and specific TaqMan assays are as follows: ABCB1- Hs00184500_m1; ABCC1- Hs00219905_m1; ABCG2- Hs01053790_m1; LRP1- Hs00233856_m1, and Hs99999905_m1 for glyceraldehyde-3-phosphate dehydrogenase; GAPDH (Thermo Fisher Scientific Waltham, MA, USA) were used to assess RNA expression according to the manufacturers’ instructions. We added 3 μL of three-times-diluted RT products to a single real-time polymerase chain reaction (RT-PCR). All the reactions were performed in triplicate in 96-well plates under the following thermal cycling conditions: 5 min at 95 °C followed by 40 cycles of 10 s at 95 °C and 30 s at 60 °C. The reactions run in the Optical Real-Time PCR Thermocycler (Biometra GmbH, Göttingen, Germany), and the threshold cycle data (Ct) were collected using qPCRsoft (Biometra GmbH, Göttingen, Germany). For the relative quantification (RQ), the samples were normalized against the expression of GAPDH mRNA using the ΔΔCT method.

### 2.6. Immunocytochemical Evaluation of MTCO-1 and 8-OHdG

Immunocytochemical staining was applied to assess the semiquantitative expression and distribution of MTCO-1 (Cytochrome c oxidase subunit I) and 8-OHdG (8-hydroxy-2’-deoxyguanosine) in the resistant and sensitive gastric cancer cells after normal and simulated microgravity with doxorubicin. Cells underwent protocols as described in Section 2.2. The cells after the treatment were seeded on 10-well slides (Thermo Fisher Scientific, Waltham, MA, USA) and incubated for 48 h. After this time, cells were washed with PBS, fixed in 4% PFA (paraformaldehyde), and washed with PBS. Next, the immunocytochemical assay Expose Mouse, and Rabbit Specific HRP/DAB Detection IHC kit (Abcam, Cambridge, UK, ab80436) was used. In brief, after washing with PBS (2 × 5 min), peroxidase activity was blocked by 30 min incubation with 1% H_2_O_2_; then, samples were permeabilized by incubation with 1% Triton X-100 (Sigma, Poznan, Poland) in PBS (BioShop, Labempire, Rzeszow, Poland). Later, cells were incubated with monoclonal antibodies against MTCO-1 (1:200; #459600, Invitrogen Warsaw, Poland) and 8-OHdG (1:200, sc-66036, Santa Cruz, Heidelberg, Germany) for 24 h at 4 °C. Then, cells were incubated with secondary horseradish peroxidase (HRP) conjugated antibody. The samples were then incubated with a mixture of diaminobenzidine-H_2_O_2_ to show the HRP marker and were counterstained with hematoxylin (Roth, Warsaw, Poland) for 3 min. After dehydration in a gradient of ethanol (Chempur, Piekary Śląskie, Poland) and xylene (Chempur, Piekary Śląskie, Poland), the microscope slides were covered with DPX (Sigma-Aldrich, Poznan, Poland). A straight microscope (Olympus BX53, Tokyo, Japan) was used for sampling. The number of stained cells was determined by counting 100 cells in three randomly selected fields. First, the staining of the cells was tested. The percentage of stained cells was shown in the table, after which the intensity of the staining was estimated. Qualitative scoring was used for the intensity assessment of the immunohistochemical staining and was classified as follows: (−) negative (no reaction), (+) weak, (++) moderate, and (+++) strong [18].

## 3. Results

**Figure 2:**
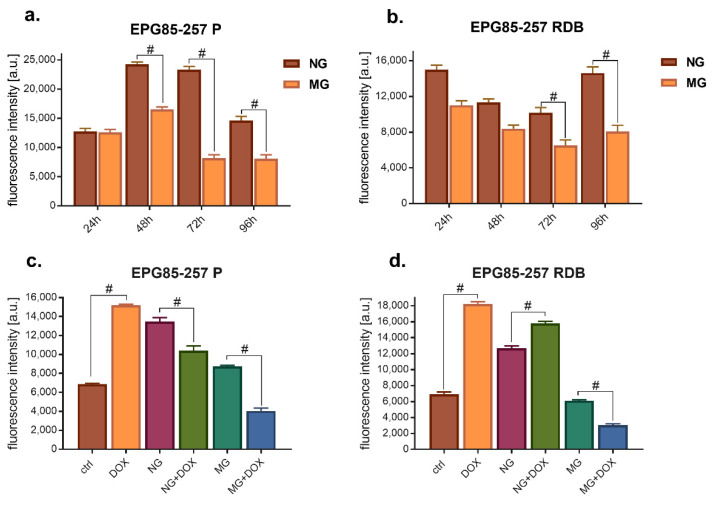
Cells’ viability of gastric cancer cells (**a**) sensitive and (**b**) drug-resistant after different exposure times to microgravity in the range of 24–96 h; the effects of the 72 h exposure to MG or NG with doxorubicin (+DOX) on the viability of (**c**) sensitive, and (**d**) resistant cells; (C_DOX_ = 0.5 ng/mL), # *p* < 0.05.

This study determined the effect of altered gravity in gastric cancer cells. Firstly, the effects of simulated microgravity (MG) in relation to normal gravity (NG) on the proliferation of cancer cells with and without drug resistance (EPG85-257 RDB, EPG85-257 P) were assessed (Figure 2). The obtained results indicate that microgravity reduces ca. 3× cell viability in sensitive cells (EPG85-257 P) and 4× decrease vs. NG alone in the case of resistant cells. Furthermore, the combination with doxorubicin caused a significant cell viability decrease, particularly in resistant cells. Interestingly, normal gravity alone and with DOX stimulated cell viability.

**Figure 3:**
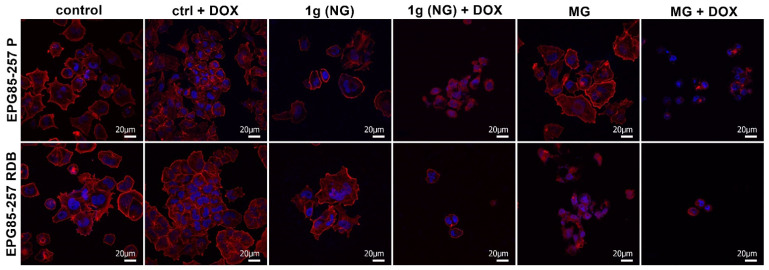
Assessment of the actin cytoskeleton in sensitive (P) and resistant (RDB) human gastric cancer cells exposed 72 h to normal or micro-gravity in the presence of doxorubicin (DOX = 0.5 ng/mL) compared to controls. NG—normal gravity; MG—microgravity.

On the other hand, studies performed with confocal microscopy showed significant changes in the cytoskeleton of the examined cells subjected to NG or MG with doxorubicin (Figure 3). The changed shape of cells, absence of lamellipodia, and intracellular reorganization of cytoskeleton fibers (F-actin) were observed after 72 h exposure to simulated microgravity, particularly with doxorubicin. Remarkably, resistant cells were affected stronger than sensitive ones. We could also observe a reduced number of cells after MG + DOX in both cell lines and significantly reduced and shrunken EPG85-257 RDB cells after NG + DOX, MG, and MG + DOX.

**Figure 4:**
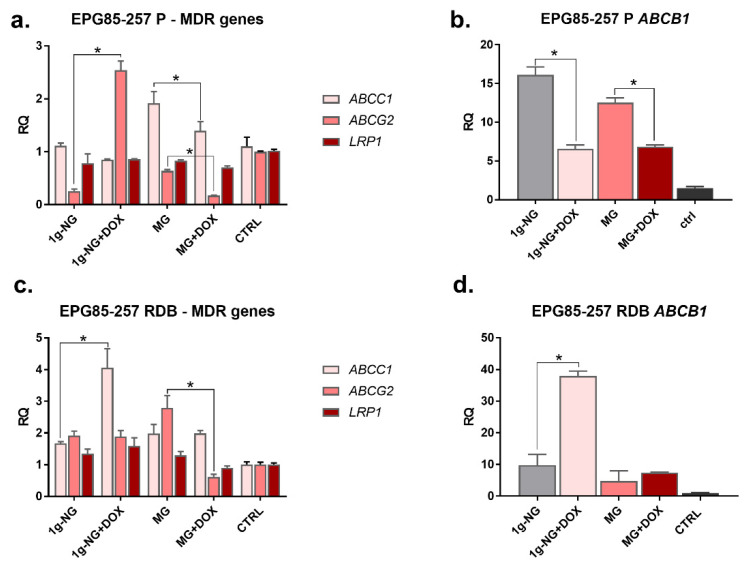
The assessment of resistance genes expression in human gastric cancer cells sensitive EPG85-257 P (**a**,**b**) and resistant EPG85-257 RDB (**c**,**d**) exposed 72 h to microgravity in the presence of doxorubicin (DOX, 0.5 ng/mL). Genes: *ABCB1*—P-glycoprotein; *ABCC1*—MRP1; *ABCG2*—BCRP; *LRP1*—LDL receptor-related protein 1. NG—normal gravity; MG—microgravity, CTRL—cells cultivated without centrifugation * *p* ≤ 0.05 for samples with DOX compared to NG or MG without DOX.

Additionally, drug resistance was assessed at the gene expression level (Figure 4). We have evaluated the expression of drug resistance genes and how normal and microgravity influence their levels in human gastric cancer cells. The obtained results indicate that, under normal gravity, the expression of the ABCC1 gene increased in the EPG85-257 RDB cell line. Moreover, NG with doxorubicin stimulated the expression of ABCC1 and ABCB1 in resistant cells. In the case of the sensitive line, the expression of the ABCG2 gene increases after exposure to 20 rpm centrifugation under normal conditions (NG). In the case of 72 h exposure of cells to simulated microgravity (MG) with DOX, a reduction in the expression of ABCB1 and ABCG2 in sensitive cells vs. cells exposed to NG or MG. In the resistant cells, we observed the decrease of ABCG2 and LRP1 vs. cells exposed to MG and control cells. However, MG and MG + DOX caused decreased expression of the *ABCB1* gene in EPG85-257 RDB cells compared to NG, but not to control cells without centrifugation.

**Figure 5:**
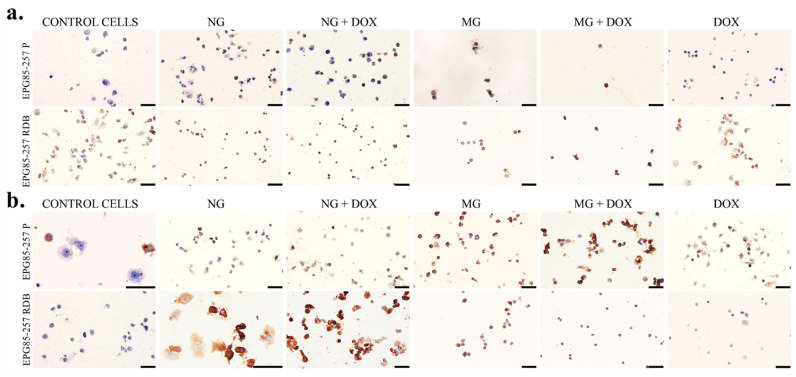
Immunocytochemical visualization of stained reaction with antibodies against (**a**) MTCO-1 (Cytochrome c oxidase subunit I) and (**b**) 8-OHdG (8-hydroxy-2’-deoxyguanosine) in human gastric cancer cells sensitive (EPF85-237 P) and chemoresistant (EPF85-237 RDB) after the 72 h exposure to normal (NG) and altered gravity (MG) with doxorubicin (DOX = 0.5 ng/mL). Scale bars correspond to 50µm.

As a next step, immunocytochemical evaluation was performed of cytochrome c oxidase subunit I (MTCO-1) and 8-hydroxy-2’-deoxyguanosine (8-OHdG) in the resistant and sensitive gastric cancer cells after normal and simulated microgravity with doxorubicin. The results of the immunostained reaction are shown in Table 1 and Figure 5a,b. In the case of MTCO-1, an increased stained immunoreaction was noted in cells exposed to NG, more intensive when combined with DOX, and the strongest reaction in 95% of cells after treatment with MG and MG + DOX. The reaction was more visible, and prominent in the resistant cells. The evaluation of the 8-OHdG immunostained reaction, which is a marker of DNA/RNA damage, revealed that the exposure to NG and NG with DOX induced stained reaction in sensitive and mostly resistant cells. The most significant and prominent DNA/RNA damage was observed after MG and MG + DOX. Additionally, we could observe shrunken cell morphology and a significantly reduced number of cells.

## 4. Discussion

So far, space flights seemed distant and only available to astronauts [19]. Recent studies indicate that microgravity can be used as a tool in anticancer therapies [20]. As it turns out, microgravity can alter the morphology of cells, especially cancerous ones. Significant changes in cell membranes, reorganization of the cytoskeleton, limited proliferation, and changes in protein and gene expression were observed [9,21]. Recent studies indicate that altered gravity induces shear stresses at the membrane surface, one of the essential factors in sensitizing cancer cells [22]. Additionally, our previous study showed morphological changes in cancer cells exposed to MG, i.e., stress fibers, membrane blebbing, and cytoskeleton reorganization [23]. Our study demonstrated that the altered gravity combined simultaneously with cytostatics (here doxorubicin) might be one of the factors modulating the cellular mechanisms and the phenomenon of drug resistance (MDR) in drug-resistant human cancer cells. We have used doxorubicin against daunorubicin-resistant cells. Doxorubicin and daunorubicin are anthracycline antibiotics, and their mechanism of action is the same. The main difference is in their interactions with cell membranes. Additionally, the resistance to anthracyclines involves the same mechanisms based on P-pg [24]. Daunorubicin reaches higher intracellular concentrations than doxorubicin [25]. Thus, we have selected DOX, a drug with weaker absorption capacity and faster cell excretion. Prasanth et al. performed a similar study with daunorubicin using leukemic and erythroleukemic cancer but incubated cells post-microgravity for 6 h. The authors observed enhanced cell migration, which indicates a reversed effect of daunorubicin. It was also observed that microgravity impacts ROS generation in a cell-type-dependent manner [9]. Chen et al. also used the gastric cancer cell model and RCCS bioreactor to simulate weightlessness. As was indicated by LC-MS, µG affected cells’ metabolism, i.e., phosphatidylethanolamine, phosphatidylcholine, arachidonic acid, and sphingosine were upregulated in µG conditions [26]. In the other study, the authors exposed lung cancer cells (A549) to simulated microgravity. It was observed that µG induced morphological abnormalities, mainly in mitochondria [27]. Ferranti et al. used a seminoma cell line (TCam-2) and exposed cells for 24 and 48 h to RPM (random positioning machine) without any additional pharmacological treatment. Comparable to our results, the authors confirmed that simulated gravity conditions modified cytoskeleton proteins, i.e., microtubules and microfilaments, strictly related to the mechanism of autophagy [28]. Additionally, after modeled microgravity conditions, Li et al. observed in MCF-7 cells revealed microfilaments were not displaying their typical radial array. MG also decreased the activity of the following kinases: FAK, PYK2, and ILK [29]. Another study, where also rotary cell culture system developed by NASA was used, proved that microgravity modulated cancer cell reaction to chemotherapy in a drug-dependent way. The authors concluded that the microgravity tool could be used in immunotherapies for space and terrestrial medicine [9]. Ahn et al. analyzed the effect of simulated microgravity (MG) in different subtypes of NSCLC (non-small cell lung cancers). Cells were rotated at 5 rpm for 48 h, and it was noted that MG promoted cell migration, and this effect was dependent on the lung cancer subtype [3]. In our study, a longer rotation time was used—72 h, and a higher rotation speed—20 rpm, and these conditions stimulated the growth of drug-sensitive cells and slightly inhibited the viability of drug-resistant cells. In this experimental setup, adding the chemotherapeutic (doxorubicin) caused a significant anticancer effect. Our results confirm that the viability of cancer cells is not affected in normal conditions, even when combined with cytostatics. We considered the obtained and literature results, and we can state that the most important in the experimental setup is speed rotation and rotation time. These parameters should be optimized individually concerning cancer type. As was demonstrated, the altered gravity strongly affects cells’ survival and cytoskeleton—related to the adhesive properties, and gene expression related to drug resistance. This effect can be used to administer the drug more efficiently because of lipid membrane reorganization and temporary unsealing.

## 5. Conclusions

The results presented in the study indicate that microgravity enhances the action of doxorubicin. An additional advantage is the anti-cancer effect of MG (μg) in the case of cells with drug resistance. As was demonstrated, microgravity conditions can modify the expression of MDR genes in gastric cancer cells. Simulated microgravity induced changes in the cancer cell, survival, and cellular cytoskeleton by making the cells more susceptible to chemotherapy. The obtained results can contribute to developing new therapeutic protocols. The study proved the antitumor potential of microgravity and its supportive role in drug delivery in gastric cancer cells. Interestingly, microgravity weakens the action of genes associated with drug resistance, which is an advantage in the case of resistance to chemotherapy. Thereby, we conclude that intracellular alterations in cell functioning in MG can also be simulated on Earth without transporting humans into space. What could this mean for medicine? This approach can be applied in medicine for cancer treatment and increase cell sensitivity to chemotherapeutics. Cosmonauts use microgravity simulators for training purposes, so perhaps they can also be used for therapeutic purposes in the near future.

## Figures and Tables

**Table 1:** Immunocytochemical evaluation of MTCO-1 and 8-OHdG in human gastric cancer cells sensitive (EPG85-257 P) and chemoresistant (EPG85-257 RDB) after 72 h exposure to normal (NG) and altered gravity (MG) with doxorubicin (DOX).

Cell Line	Sample	MTCO-1	8-OHdG
**EPG85-257 P**	**control cells**	5%, +/−	10%, ++/+
	**NG**	95%, ++	10%, +++
	**NG + DOX**	95%, ++	58%, +++/++
	**MG**	98%, ++	100%, +++
	**MG + DOX**	70%, +/++	98%, +++
	**DOX**	35%, ++	68%, ++
**EPG85-257 RDB**	**control cells**	58%, +/++	10%, +
	**NG**	95%, ++	90%, ++/+++
	**NG + DOX**	95%, ++	100%, ++/+++
	**MG**	95%, ++	100%, ++/+++
	**MG + DOX**	95%, +/++	100%, +++
	**DOX**	95%, ++	42%, ++

(−) negative (no reaction), (+) weak, (++) moderate, and (+++) strong.

## Data Availability

The data presented in this study are available on request from the corresponding author.
